# Establishing an Endoscopy Unit for Management of *Schistosoma mansoni* Hepatosplenic Morbidities in Northwestern Tanzania

**DOI:** 10.1055/a-2879-7689

**Published:** 2026-06-11

**Authors:** Humphrey Deogratias Mazigo, Crecencia Edward Chiombola, Paulina Manyiri, Kwandu Mashuda, David Charles Majinge, Tumaini Baumba, Charles Guya Mkombe, Saskia Kreibich, Christa Kasang, Antje Fuss, Andreas Mueller

**Affiliations:** 1 Medical Parasitology 150778 Catholic University of Health and Allied SciencesMwanzaTanzania, United Republic of; 2 150778 Catholic University of Health and Allied Sciences BugandoMwanzaMwanza RegionTanzania, United Republic of; 3 227206 Bugando Medical CentreMwanzaMwanza RegionTanzania, United Republic of; 4 Internal Medicine 150778 Catholic University of Health and Allied SciencesMwanzaMwanza RegionTanzania, United Republic of; 5 Internal Medicine 227206 Bugando Medical CentreMwanzaMwanza RegionTanzania, United Republic of; 6 150778 Catholic University of Health and Allied SciencesMwanzaMwanza RegionTanzania, United Republic of; 7 40130 DAHWWürzburgBYGermany; 8 40131 Medical Mission Institute WurzburgWürzburgBYGermany

**Keywords:** endoscopy upper GI tract, portal hypertension and variceal bleeding, quality and logistical aspects, training, other focus (of reviewers), epidemiology

## Abstract

**Background** In Tanzania, hepatosplenic schistosomiasis (HSS) is a common cause of morbidity and mortality among adult patients. However, capacity to manage HSS is very low. Here we report learning experience, capacity building, and collaborative partnership in establishing a functional gastrointestinal (GI) endoscopy unit.

**Methods** A need assessment for a GI endoscopy unit at Nansio District Hospital was conducted. The findings highlighted a clear need for establishing the endoscopy unit, given the burden of HSS, its associated mortality, and the lack of skilled human resources. Key priorities included capacity building for human resources, renovation of the building, provision of scholarships for residents in internal medicine, establishment of an HSS patient registry and clinic, and strengthening of ultrasound services.

**Results** The GI endoscopy unit commenced operations in August 2023. To date, a total of 666 patients have been attended (442 [66.4%] male and 224 [33.6%] female). Abdominal distension (66%), hematemesis (41%), and melena (41%) were common symptoms. Ultrasound examination revealed periportal fibrosis (PPF) in 71.5% of patients and liver image patterns C–F in 80.4%, both correlating with the severity of PPF. Of the 666 patients, 560 underwent endoscopic examination and the most common diagnosis was grade IV esophageal varices (52.1%). Among these, 84% underwent variceal ligation. On follow-up, 90% of patients achieved variceal eradication.

**Conclusion** The establishment of the GI endoscopy unit at the district hospital in rural Tanzania was possible through the support of different stakeholders.

## Background

Intestinal schistosomiasis caused by *Schistosoma mansoni* is highly endemic in Tanzania, with the northwestern region surrounding the Lake Victoria carrying almost 80% of the cases of the disease. 1 2 *Schistosoma mansoni* infection, if left untreated for long time is associated with development of the hepatosplenic disease presenting with hepatomegaly, splenomegaly and periportal fibrosis (PPF), which can cumulatively lead to portal hypertension, esophageal varices, liver surface irregularities, portal-systemic venous shunts, and hematemesis. 3 4 5 6 7 Upper gastrointestinal bleeding (UGIB) related to variceal rupture is a common complication of the portal hypertension caused by *S. mansoni* infection and is estimated to occur in 30–40% of the patients in endemic areas, 8 9 with mortality rates from a single incidence of variceal bleeding estimated at 20%. 8 In a tertiary hospital in northwestern Tanzania, 50% of adult cases with UGIB are caused by chronic *S. mansoni* morbidities 10 11 and 25% of these cases die within 6 months of diagnosis. 11 The World Health Organization (WHO) estimates that 0.2 million lives are lost every year in sub-Saharan Africa due to intestinal schistosomiasis complications, mainly bleeding esophageal varices. 12 To reduce observed mortality rates, the WHO Road map 2021–2030 recommends endemic countries to include morbidity management into their programs. 13 However, this has been a challenge due to various reasons including lack of funds and trained human resources.

Endoscopy is a key technique for the diagnosis and management of digestive system disorders including the bleeding esophageal varices related to *S. mansoni* infection portal hypertension. 14 15 16 Endoscopic management reduces mortality and improves both the quality and mental health of patients from intestinal schistosomiasis endemic communities. 17 Despite its usefulness, gastroenterology endoscopy is scarcely available in schistosomiasis endemic areas. 18 This is also evidenced by the inadequate availability of medical and technical resources to operate the endoscopy unit, especially at primary healthcare facilities. 17 19 In northwestern Tanzania, gastrointestinal endoscopy services are available at the Bugando Medical Centre, a tertiary hospital 10 and the Sekou Toure referral regional hospital, but its accessibility by poor patient from fishing communities is a challenge due to cost related to the service itself and transport. Considering the high prevalence of bleeding esophageal varices reported among fishing communities that are highly endemic for *S. mansoni* infection 10 11 and the scarcity of the human resources in the area of gastroenterology in primary healthcare facilities, 17 19 including the Nansio district hospital, there is a justification for the need to establish a gastrointestinal endoscopy unit in highly affected communities and build capacity of local healthcare workers to reduce morbidity and mortalities. 5 20 Through the support from the Else Kröner Centre for Advanced Medical and Medical Humanitarian Studies funded by the Else Kröner-Fresenius-Stiftung, capacity building and collaborative partnership of district, regional, and tertiary hospitals could be established.

## Need for gastrointestinal endoscopy service at Nansio district hospital

The district council has a total of 37 health facilities, which include one district hospital (Nansio district hospital), four health centers, and 32 dispensaries. During the period from March to December 2021 before the establishment of the gastrointestinal endoscopy, a total of 3626 admissions were recorded at the district hospital, of these 13.4% (486) had upper gastrointestinal bleeding, of these, 60.2% (293) received blood transfusion, and 14.4% (70) died. There was neither gastrointestinal endoscopy services available nor physician(s) trained on GI endoscopy. The main intervention offered to these patients was blood transfusion to stabilize them for referral either to Bugando Medical Centre or Sekou Toure hospitals located on mainland. Anesthesiology and radiology services (mostly for orthopedics and pulmonary/chest X-rays) were available. The Nansio district hospital has X-rays machine and surgical services are provided. Considering the geographical location of the district, with transport challenges, untrained staff and lack of the endoscopy services compounded by high number of UGIB patients and high number of referrals, there was a justification to establish the GI endoscopy unit at this facility.

The Ukerewe district council is made up of 38 islands distributed on Lake Victoria, of these, 15 islands have permanent residents and 23 are fishing camps. According to population census of 2022, the district had a total of 387,815 inhabitants. 21 The district consists of a total of 77 villages, 520 subvillages, and 25 wards spread across four divisions, including Mumbuga, Mumlambo, Ilangala, and Ukara. The common economic activities of the inhabitants are fishing and subsistence farming (cultivating mainly cassava, maize, banana, potatoes, and a variety of fruits). Lake Victoria is the main source of water for domestic use, recreation and agriculture.

The district is endemic for intestinal schistosomiasis, and *S. mansoni* infection has been recorded in preschool children, 22 school aged children 23 and adults. 3 Hepatosplenic disease related to *S. mansoni* infection characterized by PPF, hepatomegaly, splenomegaly, and hepatosplenomegaly are common on the island. 7 23 24 The recent study reported the prevalence of splenomegaly, PPF, hepatomegaly, and portal vein dilation of 40.5%, 48.1%, 66.2%, and 67.7% at community level. 5 The routine control of schistosomiasis done by the National NTD program targets only school aged children within the school environment and all those who are out of school are not included. The Else Kröner Center for Advanced Medical and Medical Humanitarian Studies funded by the Else Kröner Centre for Advanced Medical and Medical Humanitarian Studies is supporting mass drug administration campaigns targeting adult communities in 30 highly endemic villages. 25

## Establishment of Gastrointestinal Endoscopy Unit

**Fig. 1:**
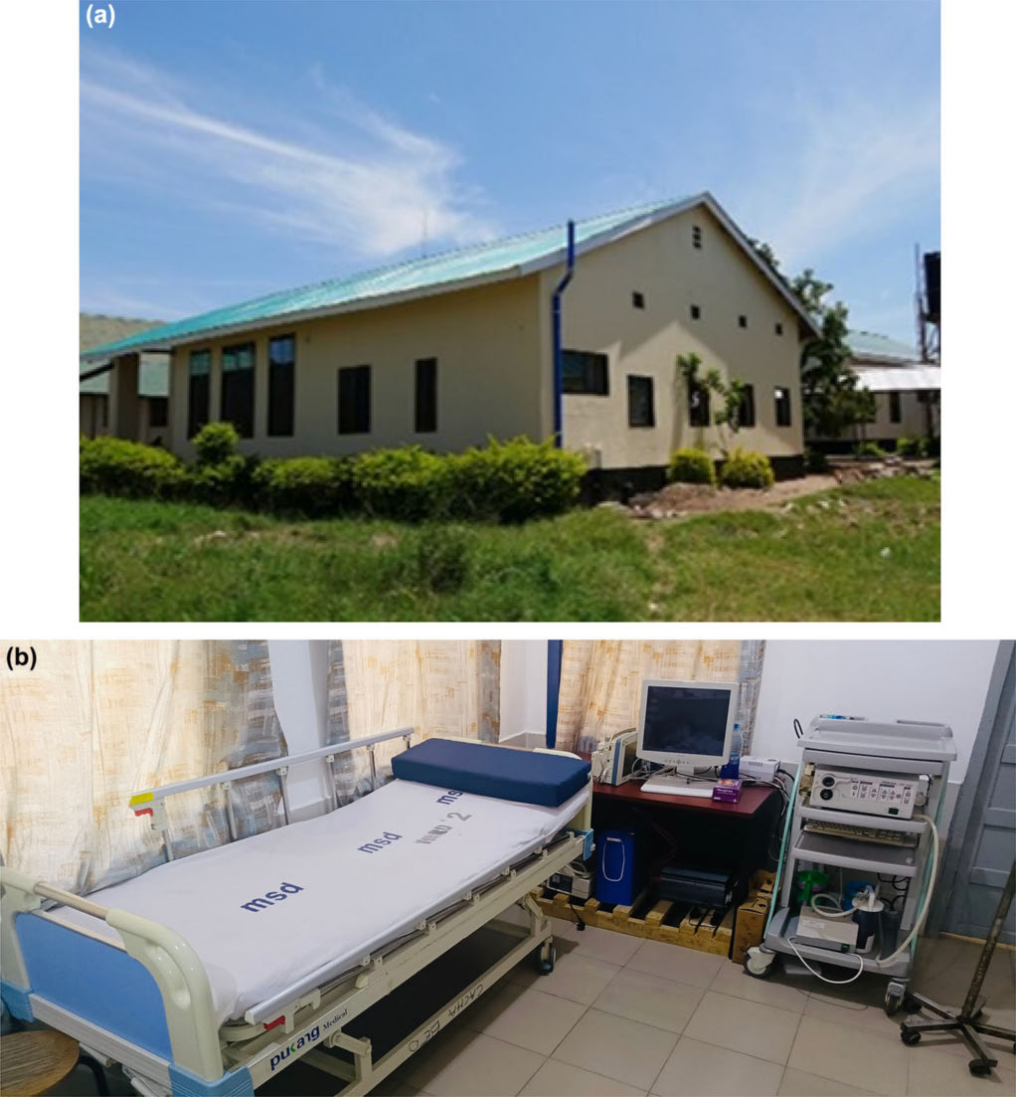
The endoscopy unit at Nansio district hospital. ( **a** ) Endoscopy building. ( **b** ) Endoscopy machine installed at the building.

The Nansio District Hospital Management team and the district authority offered the old theatre building to be used for the establishment of the GI endoscopy unit. The building needed substantial renovation including the roofing materials, ceiling boards, floor and walls, establishment of endoscopy processing areas, washrooms for staff and patients, electricity and installation of wall air conditioners, and water supply ( Fig. 1 ). Following renovation, the building has a waiting area for patient, a consultation area, an endoscopy room, an endoscopy reprocessing area and recovery room with beds, washrooms for patient and staff. The unit adapted a cleaning, disinfection, reprocessing, drying, and storage protocols of the Bugando Medical Centre GI endoscopy unit.

**Fig. 2:**
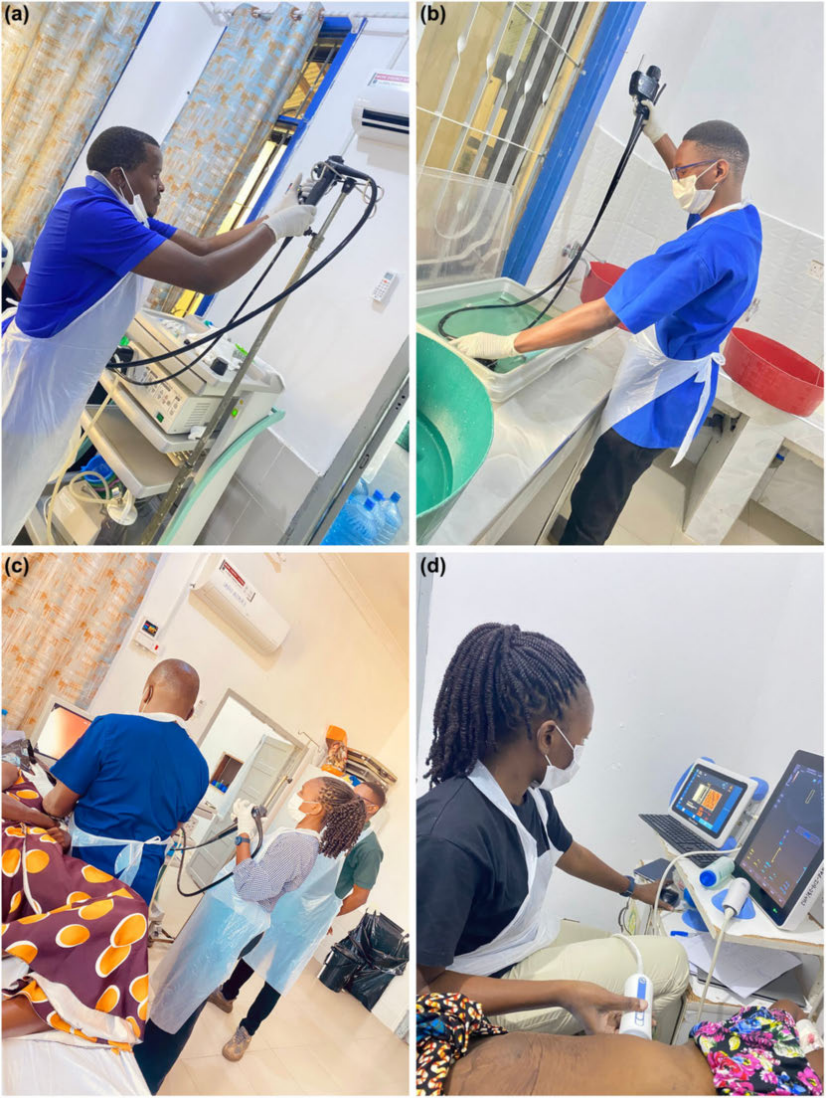
( **a, b** ) Installing the endoscopy machine and cleaning of gastroscope at the cleaning area ( **c** ). The team performing endoscopic examination on a patient. ( **d** ) A physician examining level of fibrosis using Fibroscan and Mindray Hepatus on a patient.

With support from the Medical Mission Institute and The University of Wuerzburg Medical University in Germany, the established GI endoscopy unit was equipped with one set of PENTAX EG 2990i video gastroscopes and corresponding image processor Pentax EPK-i5000 ( Fig. 2 —endoscopy machine). To ensure availability of services at all time, a second set of the same mode of endoscopy machine has been installed as a backup.

## Establishment of Partnership between District, Regional, and Tertiary

Based on the treatment guideline of Tanzania, endoscopy procedures are carried out by qualified specialized physician(s) but the Nansio District hospital so far had no qualified physician(s). To solve this problem and ensure that the unit starts offering the intended services, a partnership was established between the Catholic University of Health Sciences, Bugando Medical Centre, Sekou Toure Regional Hospital, and Nansio District Hospital, which ended up establishing a monthly outreach program lead by the Gastroenterology unit of Bugando Medical Centre. The established outreach program had the following objectives: (i) evaluating all the registered out and in patients for endoscopic diagnosis and management, (ii) conduct endoscopic examination and band ligations, (iii) training of local doctors and nurses on endoscopic process and procedures, (iv) training of local doctors and nurses to implement medical management of patients before and after endoscopic interventions, and (v) establishment of the UGIB monthly clinics in which medical services can be provided by local doctors with remote guidance from the Bugando Medical Centre and Sekou Toure gastroenterologists teams.

In the preparation for the start of the services, the following were done: (i) healthcare workers working in dispensaries and health centers were trained on how to identify patients with UGIB or at risk of vomiting blood and refer them to the district hospital and (ii) a registry of all patients with UGIB was established at the Nansio District Hospital, which allowed the local staff at Nansio hospital to register patients presenting with UGIB.

## Implementation of Endoscopic Management among Patients with Schistosomiasis-related Hepatosplenic Morbidities at Nansio Hospital Since August 2023

**Fig. 3:**
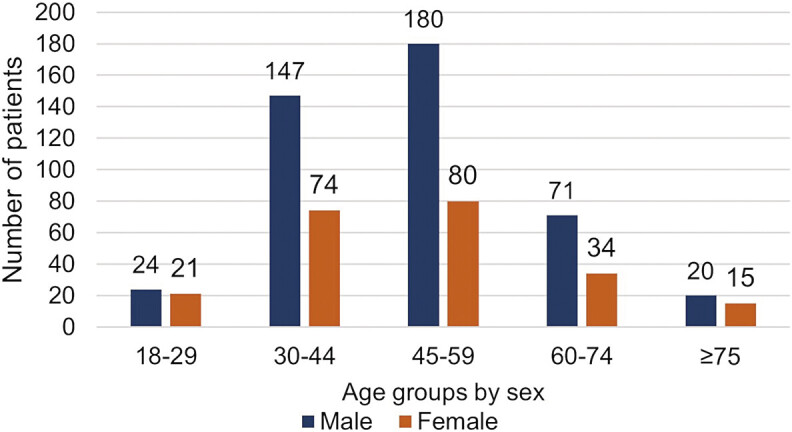
Distribution of patients managed at endoscopy unit Nansio hospital ( *n* = 666).

Endoscopy services were provided at Nansio District Hospital for 2-week periods for every 2 months to provide endoscopic services and medical management. During the visits, new patients were continuously recruited in the program. The team included two physicians (from Bugando Medical Centre and Sekou Toure Regional Hospital) who performed the endoscopic procedures. They were assisted by two experienced endoscopy nurses from the BMC endoscopy unit. For sonographic assessment, a radiologist from BMC conducted ultrasound examinations to confirm hepatosplenic morbidities on initial assessment. From Nansio District Hospital, two medical doctors and two nurses were trained at BMC and attached to the program to facilitate patient preparation, monitoring, post-endoscopy care and as part of continuous training for capacity building. In addition, two research assistants were responsible for documentation, data collection, and managing communication with patients on their follow-up visits. Following the establishment of the out-reach program and installation of the endoscopy equipment and resources, a total of 666 patients from both the community and hospital were screened for schistosomiasis-related hepatosplenic morbidities and eligibility for endoscopy. Of which, 442 (66.4%) and 224 (33.6%) were male and female, respectively, with number of males high across the age groups. The overall mean age of the patients was 48.9 ±13.6 years with majority ranging from 30 to 59 years. Fig. 3 shows patient distribution by age groups and sex.

**Fig. 4:**
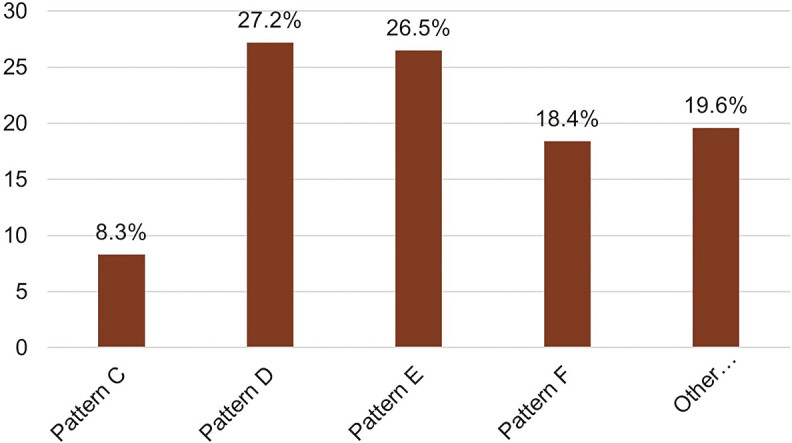
Distribution of liver parenchyma patterns from ultrasound examination ( *n* = 648).

**Fig. 5:**
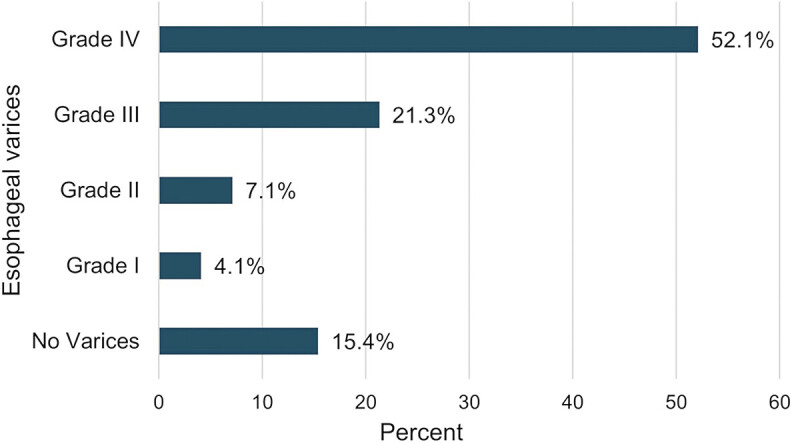
The prevalence of esophageal varices by grade in patients with hepatosplenic morbidities managed at endoscopy unit, Nansio Hospital ( *n* = 560).

The common symptoms reported by patients at the first presentation included: Abdominal distension (66%), hematemesis (41%), and melena (41%). Out of 648 patients with ultrasound results, the overall prevalence of PPF was 71.5% and 80.4% presented with liver patterns C–F which correspond to moderate to severe PPF ( Fig. 4 ). Out of 666, 560 endoscopies were performed in the unit based on clinical history and ultrasound examination. The common endoscopic findings were esophageal varices and portal gastropathy. About half of the patients presented with esophageal varices grade IV (52.1%), 21.3% with grade III, 7.1% with grade II and 4.1% with grade I ( Fig. 5 ). Out of the 560 patients who underwent endoscopy, 84% required variceal ligation and all patients with features of portal hypertension received medical management. Esophageal varices grades III and IV correlated with Liver Image C–F. Post-endoscopy care included close monitoring of patients until recovery from sedation, diet counseling, and scheduling for next appointment. The majority of the patients are currently on medical management only, following successfully achieved variceal eradication in more than 90% of the patients. In addition, patients who presented were not eligible for endoscopy management were attended by the physicians in the team accordingly. Other diagnoses were uncommon and not documented in the study records. These included patients with peptic ulcer disease and gastritis. Patients identified with these non-variceal diagnoses were channeled to the routine system of Nansio district hospital along with their results and recommendations/advice on their management from the attending physician for further management.

## Training of Local Healthcare Personnel

To ensure sustainability of the unit, it was necessary to establish short-term training to doctors, nurses and technicians on the operating process and procedures of the GI endoscopy. The short-term training involved doctors and nurses which encompasses theoretical lectures on digestive diseases and the role of GI endoscopy, hands-on training on the machine components and operating procedures, patients’ preparations, and supporting gastroenterologists during the endoscopy procedures, cleaning, disinfection, drying, and storage of the equipment. Doctors and nurses received further training on medical management of patients presenting with UGIB before and after endoscopic interventions. The team of gastroenterologists and endoscopic nurses from BMC and Sekou Toure were involved in the training process, diagnosis, and management of patients. A handbook on management of advanced stages of schistosomiasis (hematemesis and ascites) at primary healthcare was developed by the CUHAS/BMC gastroenterology unit and distributed to all primary healthcare facilities at Ukerewe district council. The handbook serves as the main resources for healthcare personnel serving communities highly affected by intestinal schistosomiasis and UGIB as the most severe and potentially lethal manifestation of the disease.

A 3 years residence scholarship in internal medicine at the Catholic University of Health and Allied Sciences/Bugando Medical Centre was offered to one of the doctors. The residence curriculum includes training in diagnostic and therapeutic endoscopy and endoscope processing. After the residence period, it is expected that she will take over as a qualified physician and run the GI endoscopy at Nansio District hospital. The mentorship and support from BMC and Sekou Toure regional hospital gastroenterology unit and our collaborating partners, medmissio—institute for Global Health and the University Hospital of Wuerzburg in Germany will continue.

## Discussion

The current report highlights the experience of establishing the endoscopy unit at a rural primary healthcare facility, capacity building of the local healthcare personnel and use of regional and tertiary hospital gastroenterology units to support, mentor and build the capacity of the district. Our approach was similar to previous gastroenterology programs. 17 The establishment of this unit has led to change in the referral practices of patients presenting with UGIB at the Nansio district hospital, with almost 99% of the cases being attended at the hospital. The establishment of the registry for patient with UGIB led to the establishment of a monthly clinic for these patients, who are attended by the local team.

The motivation of establishing the GI endoscopy unit at Nansio district hospital was based on the high prevalence of UGIB, which was mainly related to portal hypertension caused by *S. mansoni* infection requiring endoscopic interventions. 10 11 The high prevalence of PPF, hepatomegaly, splenomegaly, and portal vein dilation at community levels indicate that in these communities, the UGIB is mainly caused by *S. mansoni* infection. 3 5 7 This is supported by the available report from the tertiary hospital where 50% of the patients presenting with UGIB were suffering from *S. mansoni* infection. 10 Within 6 months of diagnosis, 11 25% of these cases had a fatal outcome. Thus, bringing endoscopy services near these communities is a health priority because it will reduce morbidity and mortalities related to UGIB, with subsequently improving the quality of life and mental health of patients. 26 The established endoscopy unit will not only be used for the management of UGIB related to *S. mansoni* infection but also for diagnosis and management of other digestive diseases such as *Helicobacter pylori* , gastritis, gastric varices, and peptic ulcers, which are common in the area. 10 Furthermore, the establishment of this unit opened a new avenue area for training residents in internal medicine from CUHAS/BMC. Moreover, the unit serves as an avenue for research which will focus on digestive diseases caused by various pathogens. Recently, elastography systems such as Echosense Fibroscan and Mindray Hepatus have been introduced into the unit to evaluate their effectiveness in detecting PPF related to *S. mansoni* infection and other causes of fibrosis such as hepatitis viruses.

Since its establishment, the unit has successfully provided services to 666 patients, predominantly males and adults from both the community and hospital registry. Male predominance is consistent with several other studies in Northwestern Tanzania where fishing is commonly conducted by men exposing them to *S. mansoni* infection and its related morbidities due to prolonged exposure to lake water (Mazigo et al., 2024, Chofle et al., 2014). On the other hand, progression of hepatosplenic disease due to *S. mansoni* infection is gradual and can take decades to occur which may explain the predominance of adults (mean = 48.9 years) among patients rather than younger people, a pattern also observed in other studies (Mazigo et al., 2024, Mueller et al., 2019, Kaatano et al., 2015). Predominance of liver parenchyma pattern C to F describes moderate to severe PPF (World Health Organization, 2000) as the cause of portal hypertension and subsequently the commonly reported symptoms, abdominal distension, hematemesis and melena. The high demand for endoscopic management in these patients is due to the high prevalence of advanced stage esophageal varices (grades III and IV), as the cause of hematemesis. Collectively, these findings describe late diagnosis for schistosomiasis-related hepatosplenic morbidities, which is clinically associated with a high risk of hematemesis and mortality (Gunda et al., 2020). Advanced hepatosplenic disease in these patients reflects the burden of chronic morbidities associated with *S. mansoni* infection in endemic regions. Furthermore, our findings underscore the importance of screening for hepatosplenic morbidities and early endoscopic management to prevent life-threating complications. Successful treatment outcomes in this program demonstrate the importance of endoscopy services as part of management in schistosomiasis-related hepatosplenic disease in endemic areas.

The sustainability of the established GI endoscopy unit at the Nansio District hospital depends on the availability of trained and qualified human resources, modern and functional equipment, and technical supports including medical supplies. 17 Structured training were planned and provided to the local team though not qualified to carry out the GI endoscopy procedures but the hands-on training and integrating the local team into the regional and tertiary hospital’s gastroenterology team provided basic training and enough exposure to the local healthcare personnel. Currently, the local team has been capacitated to run UGIB monthly clinics for patients requiring medical interventions and maintains the registry for these patients. In the future, autonomous operation of the unit is expected, after the funded resident completes her residence training on internal medicine. Conversely, it is expected that mentorship and support from regional and tertiary hospitals will continue to ensure that the unit continue to offer the intended services.

The current initiative of establishing the GI endoscopy at the district hospital has witnessed a collaborative support of different levels of hospital sharing expertise and building capacity of the lower health facilities through transferring of knowledge and working together as a team to improve patient outcomes. Bugando Medical Centre is a tertiary hospital with well-established GI endoscopy unit and a good number of physicians operating the unit, 10 whereas Sekou Toure regional hospital has a newly established unit with its personnel trained at the BMC unit. This combination and partnership is highly recommended and may serve as a model for other hospitals within the country to help transfer specialized services, from regional and tertiary hospitals to district hospital. This will not only reduce the cost of the services to the health system and users of the services but also will help to reduce morbidity and mortalities in rural communities.

In conclusion, the establishment of GI endoscopy services at the Nansio district hospital was possible with support from the Else Kröner Center for Advanced Medical and Medical Humanitarian Studies funded by the Else Kröner-Fresenius-Foundation, Germany. The partnership, mentorship, and sharing of human resources through the out-reach program between district, medical university, regional, and tertiary hospitals made the unit functional and offered the intended services. This initiative serves as an example of developing specialty and bringing specilised services to a subordinated health care level with limited capacity of diagnosing and managing digestive diseases. This in turn will reduce the number of referral from district hospital to the regional and tertiary hospitals.
